# Posicionamento da Sociedade Brasileira de Cardiologia sobre o Uso de Dispositivos Eletrônicos para Fumar – 2024

**DOI:** 10.36660/abc.20240063

**Published:** 2024-02-08

**Authors:** Jaqueline R. Scholz, Deborah Carvalho Malta, Antonio Aurélio de Paiva Fagundes, Ricardo Pavanello, Gerson Luiz Bredt, Mário de Seixas Rocha

**Affiliations:** 1 Universidade de São Paulo Hospital das Clínicas da Faculdade de Medicina Instituto do Coração SP Brasil Instituto do Coração (Incor) do Hospital das Clínicas da Faculdade de Medicina da Universidade de São Paulo (HCFMUSP), SP – Brasil; 2 Universidade Federal de Minas Gerais Belo Horizonte MG Brasil Universidade Federal de Minas Gerais (UFMG), Belo Horizonte, MG – Brasil; 3 Instituto D’Or de Pesquisa e Ensino Brasília DF Brasil Instituto D’Or de Pesquisa e Ensino (IDOR), Brasília, DF – Brasil; 4 Universidade de Brasília Brasília DF Brasil Universidade de Brasília (UNB), Brasília, DF – Brasil; 5 Hospital DFStar Brasília DF Brasil Hospital DFStar, RedeDO'r, Brasília, DF – Brasil; 6 Instituto Dante Pazzanese de Cardiologia São Paulo SP Brasil Instituto Dante Pazzanese de Cardiologia, São Paulo, SP – Brasil; 7 Universidade Estadual do Oeste do Paraná Cascavel PR Brasil Universidade Estadual do Oeste do Paraná (UNIOESTE), Cascavel, PR – Brasil; 8 Escola Baiana de Medicina e Saúde Pública Salvador BA Brasil Escola Baiana de Medicina e Saúde Pública, Salvador, BA – Brasil

**Keywords:** Sociedade Brasileira de Cardiologia (SBC)

**Table:** 

Posicionamento da Sociedade Brasileira de Cardiologia sobre o Uso de Dispositivos Eletrônicos para Fumar – 2024	
O relatório abaixo lista as declarações de interesse conforme relatadas à SBC pelos especialistas durante o período de desenvolvimento deste posicionamento, 2023/2024.	
Especialista	Tipo de relacionamento com a indústria
Antonio Aurélio de Paiva Fagundes Júnior	Declaração financeira A - Pagamento de qualquer espécie e desde que economicamente apreciáveis, feitos a (i) você, (ii) ao seu cônjuge/companheiro ou a qualquer outro membro que resida com você, (iii) a qualquer pessoa jurídica em que qualquer destes seja controlador, sócio, acionista ou participante, de forma direta ou indireta, recebimento por palestras, aulas, atuação como proctor de treinamentos, remunerações, honorários pagos por participações em conselhos consultivos, de investigadores, ou outros comitês, etc. Provenientes da indústria farmacêutica, de órteses, próteses, equipamentos e implantes, brasileiras ou estrangeiras: – Novartis, Boehringer Ingelheim, AstraZeneca, Merck Sharp & Dohme. B - Financiamento de pesquisas sob sua responsabilidade direta/pessoal (direcionado ao departamento ou instituição) provenientes da indústria farmacêutica, de órteses, próteses, equipamentos e implantes, brasileiras ou estrangeiras: – Eli Lilly and Company. Outros relacionamentos Financiamento de atividades de educação médica continuada, incluindo viagens, hospedagens e inscrições para congressos e cursos, provenientes da indústria farmacêutica, de órteses, próteses, equipamentos e implantes, brasileiras ou estrangeiras: – AstraZeneca, Merck Sharp & Dohme, Novartis.
Deborah Carvalho Malta	Nada a ser declarado
Gerson Luiz Bredt Júnior	Declaração financeira A - Pagamento de qualquer espécie e desde que economicamente apreciáveis, feitos a (i) você, (ii) ao seu cônjuge/companheiro ou a qualquer outro membro que resida com você, (iii) a qualquer pessoa jurídica em que qualquer destes seja controlador, sócio, acionista ou participante, de forma direta ou indireta, recebimento por palestras, aulas, atuação como proctor de treinamentos, remunerações, honorários pagos por participações em conselhos consultivos, de investigadores, ou outros comitês, etc. Provenientes da indústria farmacêutica, de órteses, próteses, equipamentos e implantes, brasileiras ou estrangeiras: – Novartis: Entresto; Pfizer: Eliquis; Daichii Sankyo: Lixiana; AstraZeneca: Forxiga. Outros relacionamentos Financiamento de atividades de educação médica continuada, incluindo viagens, hospedagens e inscrições para congressos e cursos, provenientes da indústria farmacêutica, de órteses, próteses, equipamentos e implantes, brasileiras ou estrangeiras: – Novo Nordisk: Ozempic. Participação em órgãos governamentais de regulação, ou de defesa de direitos na área de cardiologia: – Câmara Técnica de Cardiologia do Conselho Federal de Medicina (CFM).
Jaqueline R. Scholz	Nada a ser declarado
Mário de Seixas Rocha	Nada a ser declarado
Ricardo Pavanello	Nada a ser declarado

**Figure:**
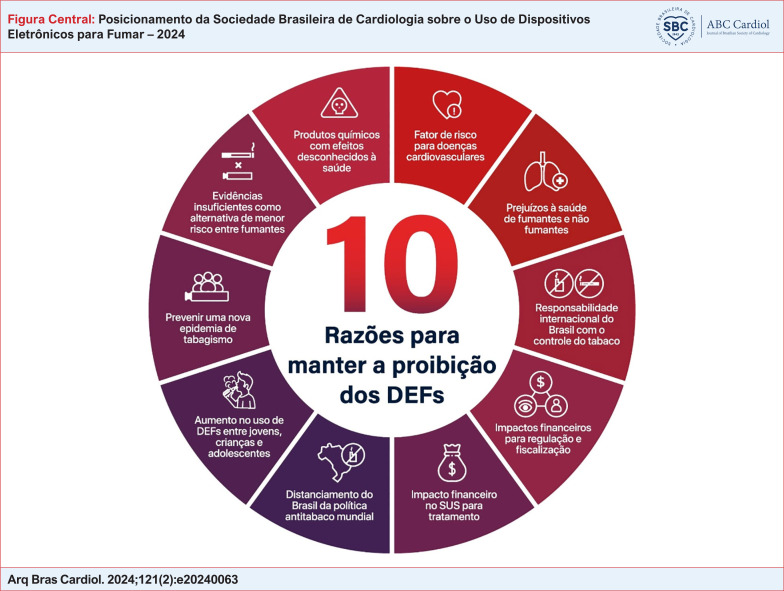
10 Razões para manter a proibição dos DEFs

A Sociedade Brasileira de Cardiologia (SBC) manifesta sua preocupação diante do atual debate acerca dos Dispositivos Eletrônicos para Fumar (DEFs), comumente conhecidos como vapes. Embora sua proibição tenha sido estabelecida em 2009 e posteriormente ratificada pela Agência Nacional de Vigilância Sanitária (ANVISA) em 2022, observa-se que a discussão sobre a comercialização, a importação e a publicidade desses dispositivos foram reavivadas em 2023 no âmbito do Senado Federal.

As alegações da indústria do tabaco, que promove os DEFs como uma alternativa de menor risco à saúde ou para a redução no consumo de cigarros convencionais, carecem de respaldo em estudos consistentes que comprovem tal teoria. Ao contrário, as evidências revelam a presença de componentes químicos prejudiciais à saúde nos DEFs, resultando em um aumento progressivo nas hospitalizações associadas aos danos pulmonares decorrentes de seu uso.^1^ Além disso, as substâncias presentes nesses dispositivos estão relacionadas ao desenvolvimento de doenças cardiovasculares, como infarto do miocárdio e acidente vascular cerebral, além de diversos outros efeitos adversos.^2–4^

Em conformidade com a proibição estabelecida pela ANVISA e pela Organização Mundial da Saúde (OMS), bem como a observação do uso desses dispositivos por jovens e não fumantes associada às evidências de malefícios decorrentes de seu consumo, a SBC considera a liberação desses dispositivos prejudicial à saúde da população. A SBC posiciona-se contrariamente à liberação dos DEFs, fundamentando tal decisão em dez razões que respaldam a manutenção de sua proibição, apresentadas a seguir (Figura Central).

## 10 Razões para Manter a Proibição

### 1. Evidências insuficientes de redução de danos entre fumantes

**Figura 1:**
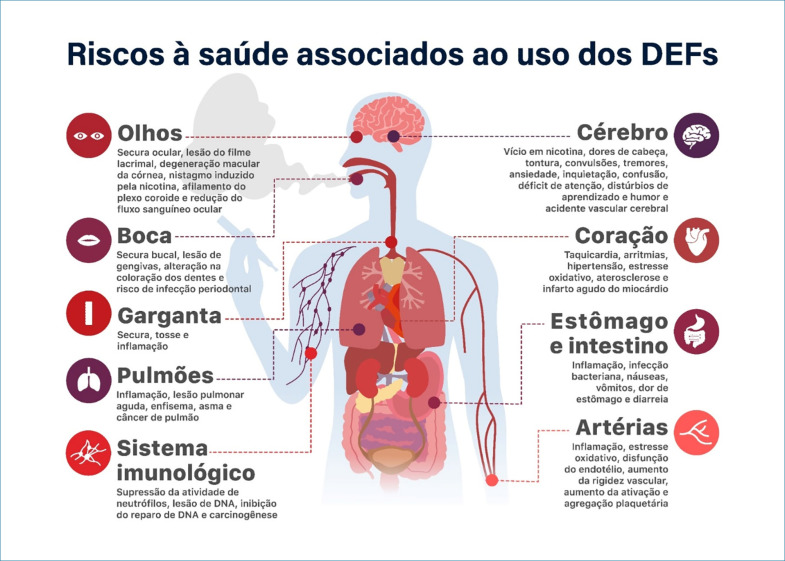
Riscos à saúde associados ao uso dos DEFs

A alegação apresentada pela indústria do tabaco de que os DEFs são uma alternativa de menor risco à saúde para substituir os cigarros convencionais carece de confirmação. Pelo contrário, estudos indicaram que jovens que fazem uso de cigarros eletrônicos têm menor propensão a cessar o tabagismo.^5,6^ Além disso, adultos fumantes que recorrem aos DEFs ou vapes exibem uma notável inclinação para a dupla utilização, que envolve cigarros tanto eletrônicos quanto regulares, o que aumenta os riscos à saúde (Figura 1).^7^ Embora o uso dual tenha sido comum nas primeiras versões dos DEFs, observa-se um crescente número de usuários exclusivos dos DEFs nas versões atuais que utilizam nicotina em freebase e sal de nicotina.^8^ Além disso, a nicotina foi identificada em usuários exclusivos de DEFs.^9^ A ausência de estudos suficientes que sustentem a tese do menor risco à saúde é relevante e contraposta por estudos clínicos e observacionais que sugerem impactos significativos na saúde dos usuários.^10–15^

### 2. O cigarro eletrônico não tem combustão, mas existem outros produtos distintos daqueles do cigarro convencional, muitos dos quais com efeitos desconhecidos na saúde humana^16^

Embora haja variações nos vapes, o produto consiste em quatro partes principais: um reservatório, um dispositivo de aquecimento, uma bateria de lítio e um bocal. No reservatório, são alojados a nicotina e, por vezes, os aromatizantes, os solventes e outros componentes químicos.^17^ A presença de solventes e aditivos, aquecidos durante o funcionamento do dispositivo, pode originar componentes tóxicos provenientes tanto dos próprios DEFs quanto de seus líquidos.^16^

Os refis e os frascos de nicotina líquida de DEFs não descartáveis representam um potencial risco de intoxicação, especialmente por ingestão acidental, absorção pela mucosa oral ou contato com a pele em caso de vazamento. Os Centros de Controle e Prevenção de Doenças dos Estados Unidos (CDC) registraram um significativo aumento nas chamadas para os centros de intoxicação relacionadas aos casos de envenenamento pelo líquido dos DEFs, sendo registrado até óbito em criança por ingestão acidental dos e-líquidos.^18^ Além disso, o descarte desses elementos representa uma séria ameaça ambiental. A quantidade de dispositivos descartados no ano de 2022 acendeu alerta das autoridades sanitárias no Reino Unido e outros países da Europa, muitos dos quais pensam em banir os dispositivos descartáveis de uso único. Eles geram toneladas de lixo eletrônico com lítio e cobre presentes nas baterias e resíduos dos e-líquidos, sendo esse conjunto de elementos considerado lixo tóxico.^19^ Adicionalmente existem também incidentes relacionados a explosões dos dispositivos.^20^

Desde 2019, tanto os CDC quanto o Food and Drug Administration (FDA) têm registrado um aumento nos casos de lesão pulmonar aguda grave associada ao uso de produtos de cigarro eletrônico ou vapes. Essa séria condição é identificada como EVALI (do inglês, e-cigarette or vaping product use-associated lung injury).^21,22^

A maioria dos pacientes diagnosticados com EVALI necessitou de hospitalização, sendo que muitos receberam cuidados intensivos e suporte respiratório. Notavelmente, 2,3% dos casos resultaram em óbito. O acetato de vitamina E, um aditivo ocasionalmente utilizado em produtos contendo tetra-hidrocanabinol (THC), está significativamente associado ao surto de EVALI. No entanto, as evidências disponíveis não são suficientes para descartar a contribuição de outros produtos químicos preocupantes.^1,23^

### 3. Vape um novo fator de risco para as doenças cardiovasculares

Diversas pesquisas indicam uma relação direta entre o uso de cigarros eletrônicos e o incremento no risco cardiovascular.^24^ A presença de nicotina nos dispositivos está vinculada a aumento da frequência cardíaca, elevação da pressão arterial e intensificação do estresse oxidativo. Além disso, o consumo regular de DEFs está associado a inflamação, disfunção endotelial, lesões vasculares e desenvolvimento de aterosclerose.^25,26^

Não apenas isso, os cigarros eletrônicos também demonstram uma relação com o aumento da probabilidade de ocorrência de infarto do miocárdio. Indivíduos que fazem uso habitual desses dispositivos apresentam uma probabilidade 1,79 vez maior de sofrer um infarto em comparação com não fumantes, conforme evidenciado em estudos.^22,27–29^

### 4. Prejuízos à saúde populacional

A comercialização e o consumo de DEFs representam uma questão de saúde pública devido ao impacto que exercem tanto sobre fumantes quanto não fumantes. Pesquisa aponta que os usuários de cigarros eletrônicos apresentam uma menor probabilidade de cessar o tabagismo de maneira voluntária, devido à significativa capacidade desses dispositivos em induzir dependência de nicotina.^30,31^

É crucial salientar a existência de usuários de DEFs que fazem uso simultâneo de cigarros convencionais, o que pode acarretar um aumento substancial no risco de desenvolvimento de doenças cardiovasculares.

Adicionalmente, a relação entre a diminuição do número de cigarros consumidos e a redução do risco à saúde não segue uma trajetória linear.^32^ Mesmo exposições a níveis reduzidos podem desencadear doenças cardiovasculares.^32^ Um estudo de caso mediu biomarcadores na urina, na saliva e no cabelo de uma família consistindo de uma gestante não fumante, seu cônjuge usuário de cigarro eletrônico e um filho do casal de 3 anos de idade. Foram identificadas elevadas concentrações de cotinina (metabólito da nicotina) e níveis significativos de metais como alumínio (associado ao enfisema pulmonar), cromo (relacionado ao câncer de pulmão), níquel (associado ao câncer de pulmão e seio nasal) e cobre (causador de danos ao fígado, rins e pulmões).^33^ No leite materno de usuárias, foram detectadas concentrações elevadas de glicerol, responsável por danos pulmonares e cardiovasculares, além de cotinina em níveis altos, assemelhando-se ao efeito da exposição do bebê ao cigarro eletrônico.^33^

### 5. Descumprimento das obrigações internacionais pelo Brasil

A introdução de novos DEFs tem conferido à indústria um espaço renovado nas discussões. A Associação da Indústria do Tabaco do Brasil já manifestou seu apoio à regulamentação, reconhecendo que a entrada de novos dispositivos no mercado é de seu interesse econômico, mesmo quando sujeitos a regulações.

É fundamental recordar que o Brasil, na qualidade de signatário da Convenção-Quadro para o Controle do Tabaco da Organização Mundial da Saúde (CQCT-OMS), está compelido, conforme disposto no artigo 5.3, a desenvolver políticas públicas de controle do tabaco resguardadas contra influências comerciais da indústria.^34^ A narrativa que a indústria está construindo, sugerindo uma busca pela redução de danos, não é uma novidade, bastando recordar os cigarros ‘light’,^35,36^ supostamente causadores de menos danos. Isso constitui meramente uma estratégia da indústria para assegurar sua permanência no mercado.

Portanto, é imperativo que o governo brasileiro esteja atento ao cumprimento de suas responsabilidades internacionais quanto ao controle do tabaco e não adote uma postura leniente em relação a essa prática.

### 6. Desafios para o cumprimento de medidas de regulação

A promulgação da Lei Antifumo no Brasil gerou significativas mudanças culturais, em especial no que se refere aos ambientes isentos de tabaco, os quais, atualmente, são respeitados por uma parcela considerável da população brasileira. O fumo em locais como aviões, restaurantes e outros espaços coletivos fechados não é mais socialmente aceito. Não obstante, é crucial compreender que a existência da Lei Antifumo representa um caminho a ser percorrido, mas não constitui uma solução integral.^34^

O Brasil enfrenta desafios decorrentes da escassez de recursos e da falta de uma fiscalização efetiva. No presente momento, a fiscalização dos DEFs (vapes) é relativamente simples, uma vez que todos os produtos dessa natureza são proibidos. Contudo, com a eventual introdução oficial de novos produtos no mercado, a regulamentação do comércio legal e o combate à falsificação, ao descaminho e ao contrabando tornar-se-ão tão ou mais complexos do que os associados ao cigarro convencional. Além disso, é necessário promover uma mudança cultural, em especial em relação ao cigarro eletrônico, que, atualmente, contraria as conquistas alcançadas no contexto do tabaco tradicional.

O Brasil tem desempenhado um papel exemplar na luta contra o tabagismo em âmbito global e as décadas de esforços resultaram em uma clara redução no consumo de tabaco, proporcionando benefícios evidentes para os indivíduos e a sociedade em geral. A proibição dos cigarros eletrônicos mantém a coerência de uma política voltada para preservar a saúde em nível tanto individual quanto coletivo.

### 7. Recursos para controle do tabaco em risco

A regulamentação dos novos produtos implicaria um ônus adicional para o orçamento da saúde, que já enfrenta restrições significativas devido a diversas prioridades. Esse desafio abrange não apenas recursos financeiros, mas também humanos, que atualmente se mostram insuficientes para supervisionar tanto o uso quanto a comercialização do cigarro convencional, bem como para desenvolver políticas eficazes de cessação do tabagismo no âmbito do Sistema Único de Saúde (SUS).

Além disso, a atenção primária e a atenção especializada enfrentam o desafio do tratamento de todas as enfermidades associadas ao consumo de produtos fumígenos, o que sobrecarrega as filas de atendimento. Considerando a insuficiência de recursos na saúde para tratar o tabagismo e suas ramificações, seria um equívoco permitir a circulação de outro produto tão prejudicial, o que certamente acarretaria um aumento nos custos com saúde no país. Um estudo conduzido em 2011 no Brasil concluiu que o custo para tratar diversas doenças crônicas decorrentes do tabagismo, no âmbito do SUS, totalizou 23,37 bilhões de reais, equivalente a 0,5% do Produto Interno Bruto e quatro vezes o montante dos impostos federais arrecadados do setor tabaco naquele ano.^37^ Esse custo tende a aumentar com a expansão do consumo de vapes. Como a maioria dos usuários é jovem, o que favorece o apelo da indústria por uma falsa percepção de segurança, estudos de curto prazo reafirmam os efeitos agudos cardiovasculares, pulmonares e cerebrovasculares e o fardo sobre o sistema de saúde certamente virá em algumas décadas. A cessação do tabagismo, inquestionavelmente, representa a estratégia mais custo-efetiva e o Brasil dispõe de um programa de tratamento eficaz, gratuito e acessível para a interrupção do tabagismo.

### 8. Diferença econômica e riscos associados na comparação entre Brasil e outros países

Comumente, a indústria dos DEFs cita países em que a comercialização do produto é liberada com o objetivo de obter sua liberação no Brasil, como um exemplo a ser seguido. Contudo, mesmo em nações com um aparato legal e regulatório mais robusto, como Estados Unidos, Austrália, Reino Unido, Nova Zelândia e França, as legislações referentes aos cigarros eletrônicos estão sendo revisadas devido ao expressivo aumento no uso de vapes entre jovens, crianças e adolescentes, com incidência em escolas de ensino fundamental.^38^ Portanto, é imprudente que o Brasil presuma que, sem as condições adequadas para garantir a aplicação integral da Lei Antifumo, seria capaz de controlar o consumo desenfreado de DEFs, expondo a população jovem aos comprovados malefícios desses produtos.

A indústria do tabaco está realizando investimentos substanciais na produção de vapes, transformando-os em um lucrativo empreendimento para as empresas internacionais do setor, que atualmente totalizam 466 marcas no mercado.^39–41^ Além disso, o produto vem sendo aperfeiçoado ao longo do tempo, oferecendo capacidade de maior oferta volumétrica dos e-líquidos nos tanques, maior concentração de nicotina e redução nos preços, favorecendo ainda mais o consumo e a adição.^42^

### 9. Proliferação dos DEFs entre jovens e não fumantes

Apesar da exposição ilegal, os adolescentes continuam a ser altamente suscetíveis aos DEFs. A Global Youth Tobacco Survey evidencia um aumento epidêmico no consumo de cigarros eletrônicos, que chega a ser três vezes superior entre adolescentes na mesma faixa etária considerando países em que a comercialização é permitida em comparação a países com o banimento da comercialização, como Brasil e Tailândia.^38^

A Pesquisa Nacional de Saúde dos Escolares,^43^ conduzida pelo Instituto Brasileiro de Geografia e Estatística e abrangendo 159.245 estudantes brasileiros, revela que a experimentação de cigarro eletrônico em algum momento da vida entre escolares de 13 a 17 anos atingiu 16,8% (IC95% 16,2-17,4), sendo que 3,6% (IC95% 3,3-4,0) utilizaram nos últimos 30 dias. Vale ressaltar que o uso de qualquer produto relacionado ao tabaco, englobando cigarros convencionais, vapes e outros, aumentou de 9% em 2015 para 12% em 2019 entre adolescentes. Portanto, após duas décadas de declínio, a tendência entre adolescentes está se revertendo, influenciada pelo uso de produtos como vapes e narguilé, conforme evidenciado pela Pesquisa Nacional de Saúde dos Escolares.^44^ Segundo a pesquisa Covitel 2023, um em cada quatro jovens entre 18 e 24 anos já experimentou cigarros eletrônicos, sendo seu uso 40 vezes mais comum na população abaixo dos 40 anos, mesmo com a venda proibida no país. Entre os usuários de cigarros eletrônicos de 15 a 24 anos, 63% nunca experimentaram cigarro convencional, indicando que os DEFs têm se tornado a forma de iniciação ao fumo na juventude.^45,46^

Mesmo sob regulamentação, a permissão da venda de DEFs apenas ampliaria as oportunidades de seu consumo entre os jovens, uma vez que o acesso seria facilitado. Além disso essa regulamentação promoveria a falsa ilusão de um produto menos nocivo.^47^ O amplo comércio, aliado à limitada capacidade de fiscalização, poderia proporcionar aos menores de idade mais chances de iniciar ou manter seu vício desde cedo, evidenciando os riscos associados à liberação do consumo de DEFs no país.^48^

### 10. Princípio da precaução

Diante das evidências disponíveis, considerando a natureza dos riscos vinculados ao uso dos novos DEFs, seu elevado potencial para adição e a incapacidade de implementação eficaz de medidas fiscalizatórias, bem como a falta de recursos destinados a tratar as consequências do consumo desses novos produtos, torna-se imperativo manter sua proibição no Brasil. Isso visa prevenir uma potencial nova epidemia de consumo de vapes ou agravamento da atual.

## Conclusão

Com base no exposto, a SBC manifesta veementemente sua oposição a qualquer regulamentação de comercialização dos DEFs, independentemente de sua modalidade.
