# Supplementary material for: Posicionamento da Sociedade Brasileira de Cardiologia sobre o Uso de Dispositivos Eletrônicos para Fumar – 2024
Source: Arq Bras Cardiol. 2024 Feb 8;121(2):e20240063. [Article in Portuguese] doi: 10.36660/abc.20240063 (PMC11081107; doi:10.36660/abc.20240063)
Supplement: Translation

# Translation: Brazilian Society of Cardiology Position Statement on the Use of Electronic Nicotine Delivery Systems – 2024

**Authors:** Jaqueline R. Scholz, Deborah Carvalho Malta, Antonio Aurélio de Paiva Fagundes, Ricardo Pavanello, Gerson Luiz Bredt, Mário de Seixas Rocha

**Affiliations:** 1 Universidade de São Paulo Hospital das Clínicas da Faculdade de Medicina Instituto do Coração SP Brazil Instituto do Coração (Incor) do Hospital das Clínicas da Faculdade de Medicina da Universidade de São Paulo (HCFMUSP), SP – Brazil; 2 Universidade Federal de Minas Gerais Belo Horizonte MG Brazil Universidade Federal de Minas Gerais (UFMG), Belo Horizonte, MG – Brazil; 3 Instituto D’Or de Pesquisa e Ensino Brasília DF Brazil Instituto D’Or de Pesquisa e Ensino (IDOR), Brasília, DF – Brazil; 4 Universidade de Brasília Brasília DF Brazil Universidade de Brasília (UNB), Brasília, DF – Brazil; 5 Hospital DFStar Brasília DF Brazil Hospital DFStar, RedeDO'r, Brasília, DF – Brazil; 6 Instituto Dante Pazzanese de Cardiologia São Paulo SP Brazil Instituto Dante Pazzanese de Cardiologia, São Paulo, SP – Brazil; 7 Universidade Estadual do Oeste do Paraná Cascavel PR Brazil Universidade Estadual do Oeste do Paraná (UNIOESTE), Cascavel, PR – Brazil; 8 Escola Baiana de Medicina e Saúde Pública Salvador BA Brazil Escola Baiana de Medicina e Saúde Pública, Salvador, BA – Brazil

**Keywords:** Brazilian Society of Cardiology (Sociedade Brasileira de Cardiologia – SBC)

**Table:** 

Brazilian Society of Cardiology Position Statement on the Use of Electronic Nicotine Delivery Systems – 2024	
The report below lists declarations of interest as reported to the SBC by the experts during the period of the development of these statement, 2023/2024.	
Expert	Type of relationship with industry
Antonio Aurélio de Paiva Fagundes Júnior	Financial declaration A - Economically relevant payments of any kind made to (i) you, (ii) your spouse/partner or any other person living with you, (iii) any legal person in which any of these is either a direct or indirect controlling owner, business partner, shareholder or participant; any payments received for lectures, lessons, training instruction, compensation, fees paid for participation in advisory boards, investigative boards or other committees, etc. From the brazilian or international pharmaceutical, orthosis, prosthesis, equipment and implants industry:- Novartis, Boehringer Ingelheim, AstraZeneca, Merck Sharp & Dohme. B - Research funding under your direct/personal responsibility (directed to the department or institution) from the Brazilian or international pharmaceutical, orthosis, prosthesis, equipment and implants industry: - Eli Lilly and Company. Other relationships Funding of continuing medical education activities, including travel, accommodation and registration in conferences and courses, from the brazilian or international pharmaceutical, orthosis, prosthesis, equipment and implants industry: - AstraZeneca, Merck Sharp & Dohme, Novartis.
Deborah Carvalho Malta	Nothing to be declared
Gerson Luiz Bredt Júnior	Financial declaration A - Economically relevant payments of any kind made to (i) you, (ii) your spouse/partner or any other person living with you, (iii) any legal person in which any of these is either a direct or indirect controlling owner, business partner, shareholder or participant; any payments received for lectures, lessons, training instruction, compensation, fees paid for participation in advisory boards, investigative boards or other committees, etc. From the brazilian or international pharmaceutical, orthosis, prosthesis, equipment and implants industry: - Novartis: Entresto; Pfizer: Eliquis; Daichii Sankyo: Lixiana; AstraZeneca: Forxiga. Other relationships Funding of continuing medical education activities, including travel, accommodation and registration in conferences and courses, from the brazilian or international pharmaceutical, orthosis, prosthesis, equipment and implants industry: - Novo Nordisk: Ozempic. Participation in government-related regulatory authorities or advocacy authorities in cardiology: - Cardiology Technical Chamber of the Federal Council of Medicine (In portuguese: Conselho Federal de Medicina - CFM)
Jaqueline R. Scholz	Nothing to be declared
Mário de Seixas Rocha	Nothing to be declared
Ricardo Pavanello	Nothing to be declared

**Figure:** 10 Reasons for supporting prohibition on ENDS

The Brazilian Society of Cardiology (SBC) manifests its concern about the current debate on Electronic Nicotine Delivery Systems (ENDS), commonly known as vapes. Although their prohibition in Brazil was established in 2009 and later confirmed by the Brazilian Health Surveillance Agency (ANVISA) in 2022, the discussion on the sales, importation, and advertising of those devices was revived in 2023 in the Brazilian Federal Senate.

The claims by the tobacco industry that, as compared to conventional cigarettes, ENDS are an alternative of lower health risk or reduce the consumption of conventional cigarettes have not been supported by consistent studies. On the contrary, there is evidence that ENDS contain harmful chemical components, which results in a progressive increase in the number of hospitalizations related to lung damage due to their use.^1^ In addition, substances present in those devices are related to the development of cardiovascular diseases, such as myocardial infarction and stroke, as well as to several other adverse effects.^2–4^

In accordance with the prohibition of ENDS by the ANVISA and the World Health Organization (WHO), in addition to their well-known use by young individuals and non-smokers compounded by the evidence of harm due to their consumption, the SBC considers the legalization of ENDS harmful to the population's health. Thus, the SBC opposes their legalization and has based the decision on maintaining ENDS prohibition on the following ten reasons (Central Illustrantion).

## 10 Reasons for Supporting Prohibition

### 1. Insufficient evidence to support harm reduction among smokers

The allegation by the tobacco industry that ENDS are a lower health-risk alternative to replace conventional cigarettes still lacks confirmation. On the contrary, some studies have indicated that young users of electronic cigarettes (e-cigarettes) are less likely to quit smoking.^5,6^ In addition, adult smokers who resort to ENDS or vapes have a remarkable tendency to dual use, which involves both e-cigarettes and regular cigarettes, thus, increasing health risks (Figure 1).^7^ Although dual use was common with the first versions of ENDS, a growing number of exclusive ENDS users has been observed with the current versions that contain freebase nicotine and nicotine salt.^8^ Furthermore, nicotine has been identified in exclusive ENDS users.^9^ The absence of sufficient studies to support the thesis of lower health risk is relevant and compounded by clinical and observational studies that suggest significant impacts on the users’ health.^10–15^

**Figure 1:** Risks to health associated with ENDS use

### 2. E-cigarettes involve no combustion, but contain other products different from those of conventional cigarettes, many of which with unknown effects on human health^16^

Although vapes vary, they consist of four major parts: a reservoir or cartridge, a heating element, a lithium battery, and a mouthpiece. The reservoir contains nicotine and, sometimes, flavorings, solvents, and other chemicals.^17^ The presence of solvents and additives, heated during the vaping process, might originate toxic components from both the ENDS and their liquids.^16^

The refills and liquid nicotine bottles of non-disposable ENDS represent a potential risk of intoxication, especially through accidental ingestion, absorption through the oral mucosa, or contact with skin in case of leakage. The North American Centers for Disease Control and Prevention (CDC) have registered a significant increase in the number of calls to intoxication centers related to poisoning with the ENDS liquids, and even child death due to accidental ingestion of e-liquids.^18^ In addition, the disposal of those elements is a serious environmental hazard. The number of devices discarded in 2022 raised an alert among the sanitary authorities of the United Kingdom and other European countries, many of which even consider banning single-use disposable devices. The devices generate tons of electronic waste with lithium and copper, present in the batteries, and residues from the e-liquids, and this set of elements is considered toxic waste.^19^ Furthermore, incidents related to the explosion of ENDS have been reported.^20^

Since 2019, both the CDC and the Food and Drug Administration (FDA) have identified an increase in the number of cases of severe acute pulmonary damage associated with the use of e-cigarettes or vapes. This severe condition is known as ‘e-cigarette or vaping product use-associated lung injury’ (EVALI).^21,22^

Most patients diagnosed with EVALI have required hospitalization and many have received intensive care and respiratory support. It is worth noting that 2.3% of the cases have resulted in death. Vitamin E acetate, an additive occasionally used in tetrahydrocannabinol (THC)-containing products, is significantly associated with the EVALI outbreak. However, the evidence available is not sufficient to rule out the contribution of other chemicals of concern.^1,23^

### 3. Vape – a new risk factor for cardiovascular diseases

Several studies have indicated a direct relationship between the use of e-cigarettes and the increase in cardiovascular risk.^24^ The presence of nicotine in the devices is linked to an increase in heart rate and in blood pressure, as well as oxidative stress intensification. In addition, the regular consumption of nicotine is associated with inflammation, endothelial dysfunction, vascular lesions, and development of atherosclerosis.^25,26^

Not only that, but e-cigarettes have shown to be related to an increased likelihood of myocardial infarction. Individuals who use those devices regularly are 1.79 time more likely to have an infarction as compared to non-smokers, as evidenced by some studies.^22,27–29^

### 4. Harm to population health

The sales and consumption of ENDS represent a public health issue because of their impact on both smokers and non-smokers. A study has shown that e-cigarette users are less likely to quit smoking willingly because those devices significantly induce nicotine dependence.^30,31^

It is crucial to emphasize the existence of individuals using simultaneously ENDS and conventional cigarettes, which can substantially increase their risk for cardiovascular diseases.

In addition, the relationship between the reduction in the number of cigarettes consumed and the reduction in health risk does not follow a linear trajectory.^32^ Even the exposure to reduced levels can trigger cardiovascular diseases.^32^ A case study has measured biomarkers in the urine, hair, and saliva of a family unit consisting of an e-cigarette-user, a pregnant non-smoker, and their 3-year-old child. That study has shown elevated concentrations of cotinine (a nicotine metabolite) and significant levels of metals, such as aluminum (associated with pulmonary emphysema), chrome (related to lung cancer), nickel (associated with lung and nasal sinus cancer), and copper (harmful to the liver, kidneys, and lungs).^33^ The breast milk of users has shown elevated concentrations of glycerol, responsible for pulmonary and cardiovascular injuries, as well as high levels of cotinine, similarly to the effect of the baby's exposure to e-cigarette.^33^

### 5. Brazil's non-compliance with international obligations

The introduction of new ENDS has provided the tobacco industry with a renewed space in the discussions. The Brazilian Tobacco Industry Association has manifested its support to regulation, recognizing that the entry of new devices in the market is of its economic interest even when subjected to regulations.

It is fundamental to recall that Brazil, as a signatory of the WHO's Framework Convention on Tobacco Control (FCTC), is compelled, as stated in Article 5.3, to develop public policies for tobacco control protected against the industry's commercial influences.^34^ The tobacco industry's narrative, which suggests they are searching for tobacco harm reduction, is not new, just to remember the ‘light’ cigarettes,^35,36^ claimed by that industry to be less harmful. That is simply an industry strategy to ensure its permanence in the marketplace.

Thus, it is imperative that the Brazilian government be attentive to comply with its international responsibilities for tobacco control and not adopt a lenient attitude toward it.

### 6. Challenges for complying with regulatory measures

Enactment of the Anti-smoking Law in Brazil has caused significant cultural changes, especially regarding the smoke-free environments, currently respected by a significant part of the Brazilian population. Smoking in places, such as airplanes, restaurants, and other enclosed spaces, is no longer socially accepted. However, it is crucial to understand that the Anti-smoking Law represents a path to be travelled, not a complete solution.^34^

Brazil faces challenges due to the scarcity of resources and ineffective law enforcement. At the moment, ensuring law enforcement on ENDS should be relatively simple, because all such products are forbidden. However, with the occasional official introduction of new products in the marketplace, the regulation of legal sales, as well as fighting counterfeiting and smuggling, would become as complex as or even more complex than those associated with conventional cigarettes. In addition, it is necessary to promote a cultural change, especially regarding e-cigarettes, which currently challenge the achievements in the context of traditional tobacco.

Brazil has played an exemplary role in the fight against smoking in the global context and the decades of struggle have resulted in a clear reduction in tobacco consumption, yielding evident benefits for individuals and society in general. Prohibiting e-cigarettes maintains the coherence of a policy to preserve health at both individual and collective levels.

### 7. Resources for tobacco control at risk

The regulation of new products would burden the health budget, which already faces significant restrictions due to other priorities. This challenge involves not only financial resources, but human as well, which are currently insufficient to ensure control of both the use and the sale of conventional cigarettes, as well as to develop effective policies for smoking cessation at the Brazilian Unified Health System (SUS) level.

Furthermore, primary and specialized healthcare faces the challenge of treating all diseases associated with the use of tobacco products, which overloads the waiting lists for healthcare. Considering the scarcity of health resources to treat smoking addiction and its consequences, it would be a mistake to allow the entry of another harmful product that would certainly increase healthcare costs in Brazil. A study conducted in 2011 in Brazil concluded that the cost of treating, in the SUS, several chronic diseases due to tobacco use added up to 23.37 billion reais, equivalent to 0.5% of the gross domestic product and four times the amount of the federal taxes collected from the tobacco sector in that year.^37^ That cost tends to increase with the expansion of the use of vapes. Considering that most vape users are young, which favors the industry's appeal due to a false perception of safety, short-term studies have confirmed the acute cardiovascular, pulmonary, and cerebrovascular effects, and the consequent burden on the healthcare system will certainly be evidenced in a few decades. Smoking cessation, undoubtedly, represents the best cost-effective strategy and Brazil has an effective, free, and accessible treatment program for smoking cessation.

### 8. Economic differences and associated risks in comparing Brazil to other countries

Commonly, the ENDS industry cites countries where the sales of those products are permitted aiming at convincing Brazil to allow them, as an example to be followed. However, even in nations with a more robust legal and regulatory apparatus, such as the United States, Australia, United Kingdom, New Zealand, and France, the legislation on e-cigarettes is being reviewed because of the significant increase in the use of vapes among young individuals, children, and adolescents, including in elementary schools.^38^ Thus, it would be reckless to assume that, without the proper conditions to ensure the total Anti-smoking Law enforcement, Brazil could control the unrestrained consumption of ENDS, and this exposes its young population to the proven harm of those products.

The tobacco industry is investing substantially in the production of vapes, transforming them into a profitable product for the international companies in the sector, which currently add up to 466 brands in the marketplace.^39–41^ In addition, the product has been improved over time, with greater e-liquid volume capacity in the tanks, higher nicotine concentration, and price reduction, favoring consumption and addiction.^42^

### 9. Proliferation of ENDS use among young individuals and non-smokers

Despite the illegal exposure, adolescents remain highly susceptible to ENDS. The Global Youth Tobacco Survey has evidenced an epidemic increase in the consumption of e-cigarettes, which tends to be three times higher among adolescents of the same age group in countries where the ENDS sales are permitted as compared to those where the ENDS sales have been banned, such as Brazil and Thailand.^38^

The Brazilian National Survey of Schoolchildren Health,^43^ conducted by the Brazilian Institute of Geography and Statistics, involving 159 245 Brazilian schoolchildren, has shown that up to 16.8% (95% CI, 16.2-17.4) of those aged 13-17 years had experimented with e-cigarettes at a certain time, and 3.6% (95% CI, 3.3-4.0) had used it in the preceding 30 days. It is worth noting that the use of any tobacco-related product, comprising conventional cigarettes, vapes, and others, increased from 9% in 2015 to 12% in 2019 among adolescents. Thus, after two decades of decline, the trend among adolescents is reversing, influenced by products, such as vapes and waterpipes, as evidenced in the Brazilian National Survey of Schoolchildren Health.^44^ According to the Covitel study 2023, one in every four young individuals aged 18-24 years had already experimented with e-cigarettes, and their use was 40 times more common in the population under 40 years of age, even with their sales forbidden in Brazil. Of the users of e-cigarettes aged 15-24 years, 63% had never experimented with conventional cigarettes, indicating that ENDS have become the gateway to smoking among the youth.^45,46^

Even under regulation, the permission to sell ENDS would only widen the opportunities for their consumption among youngsters, considering that their access to the product would be facilitated and the misleading conception of a less harmful product would be promoted along with its regulation.^47^ The widespread sales, associated with the limited capacity of effective enforcement, could provide the youngsters with more chances to initiate or maintain their addiction since an early age, which evidences the risks associated with the legalization of ENDS consumption in Brazil.^48^

### 10. Precautionary principle

In face of the evidence available and considering the nature of the risks related to the use of new ENDS, their high potential for addiction, and the inability to effectively implement control measures, as well as the lack of resources destined to handle the consequences from the use of new products, it is imperative to maintain their prohibition in Brazil. This is aimed at preventing a potential new epidemic of vaping or the worsening of the ongoing one.

## Conclusion

In conclusion, the SBC strongly opposes the regulation of the sales of ENDS of all modalities.
